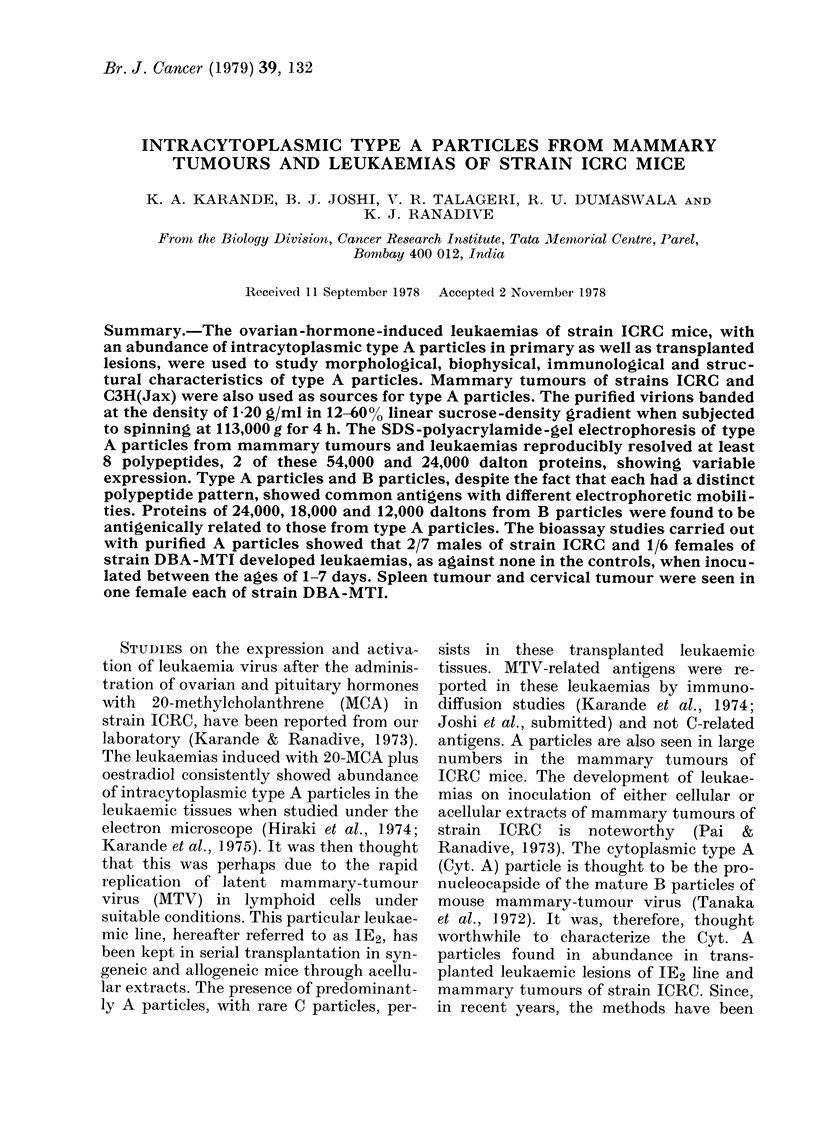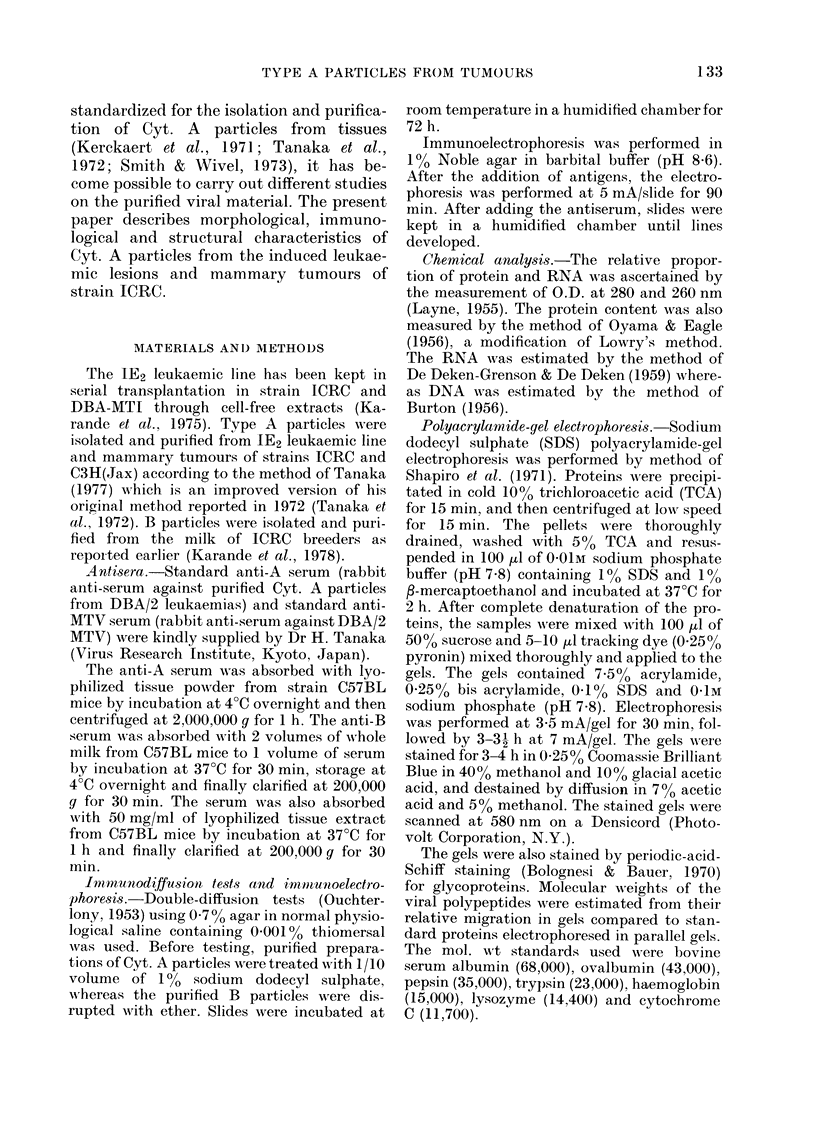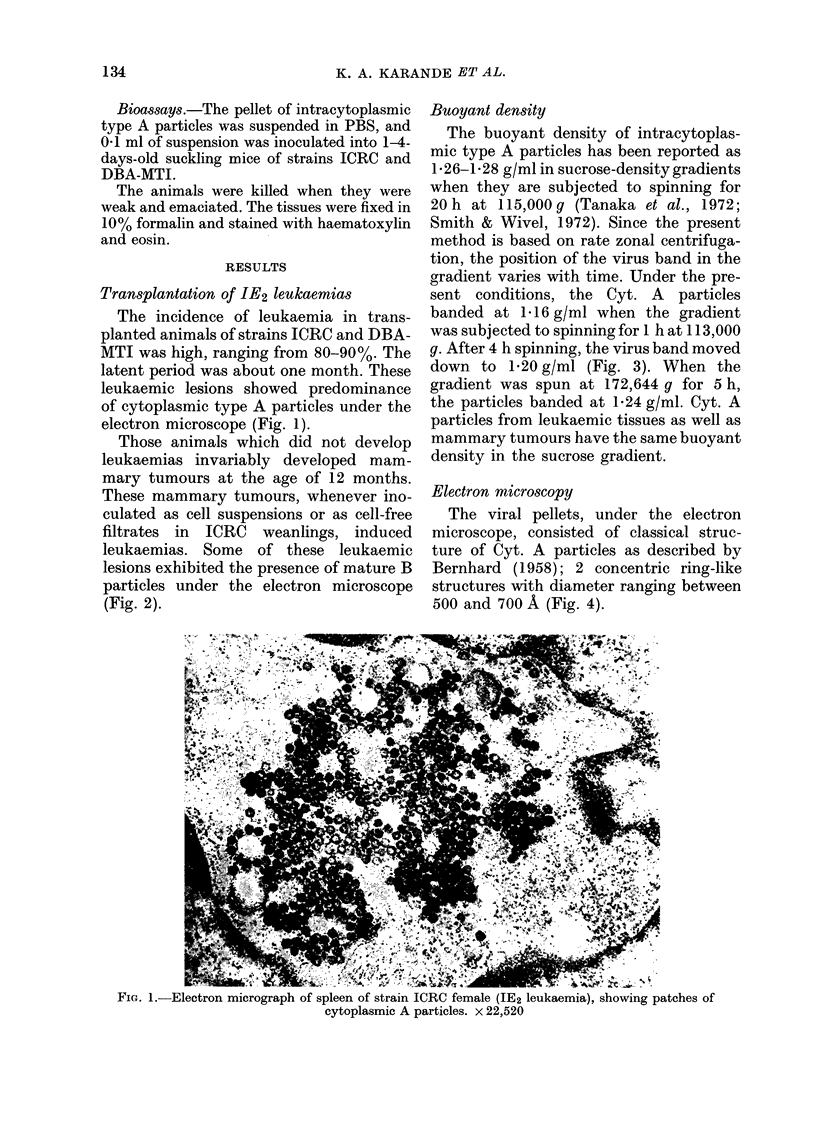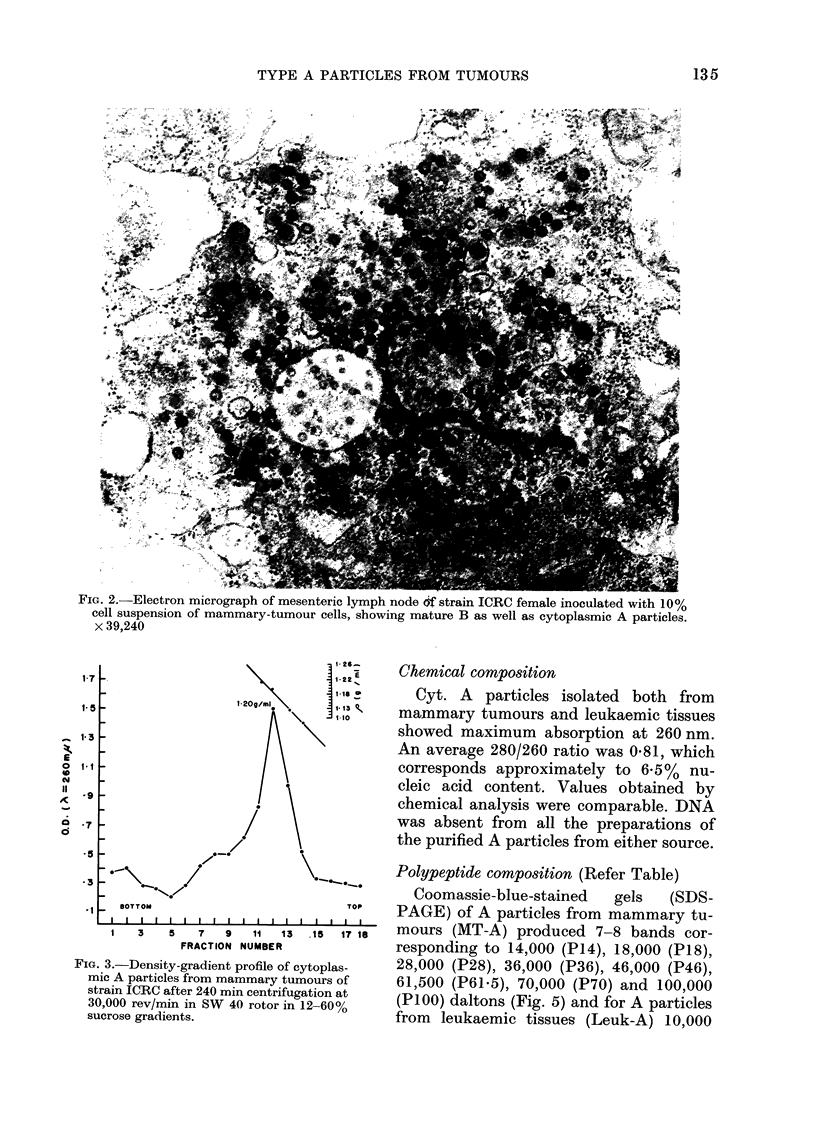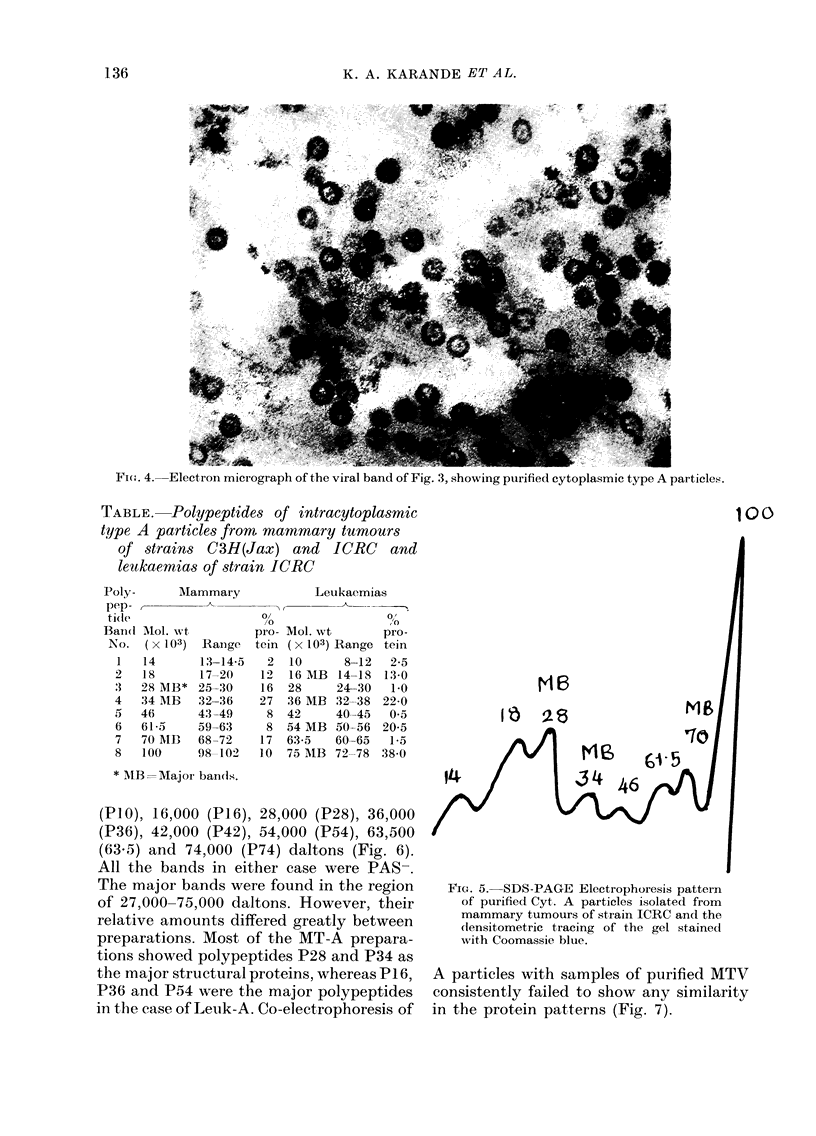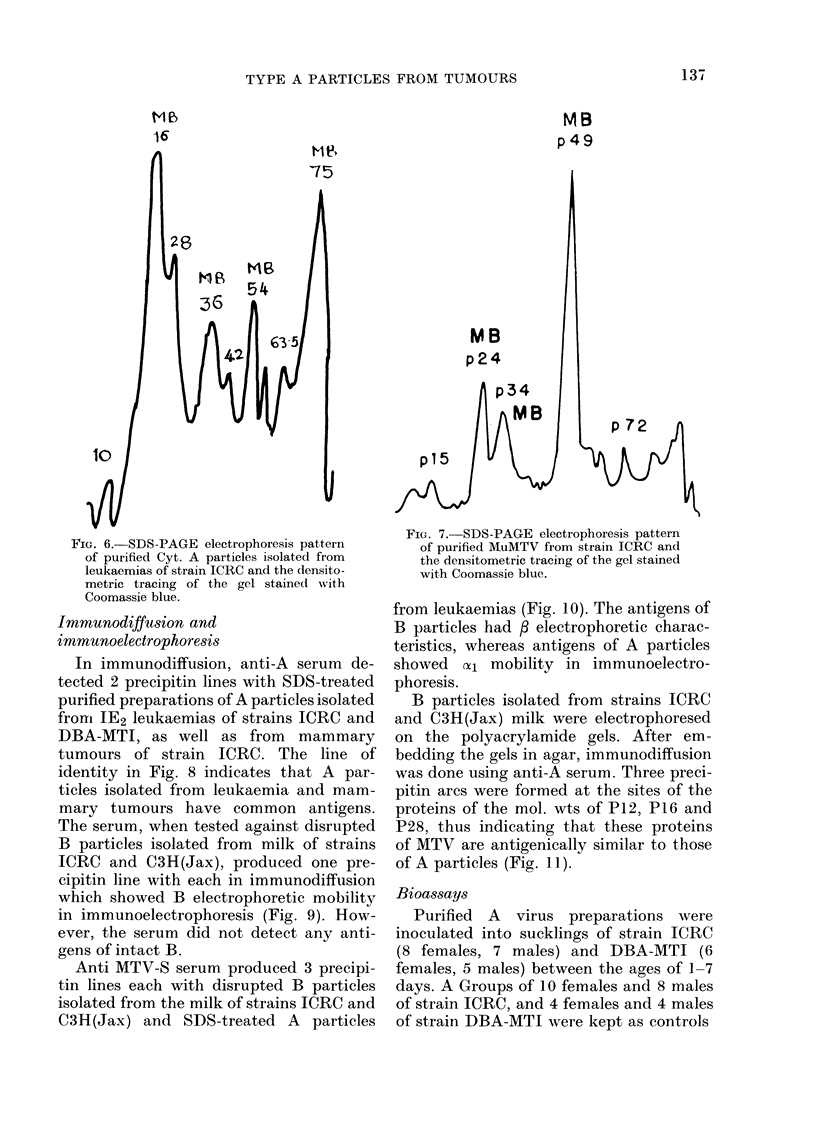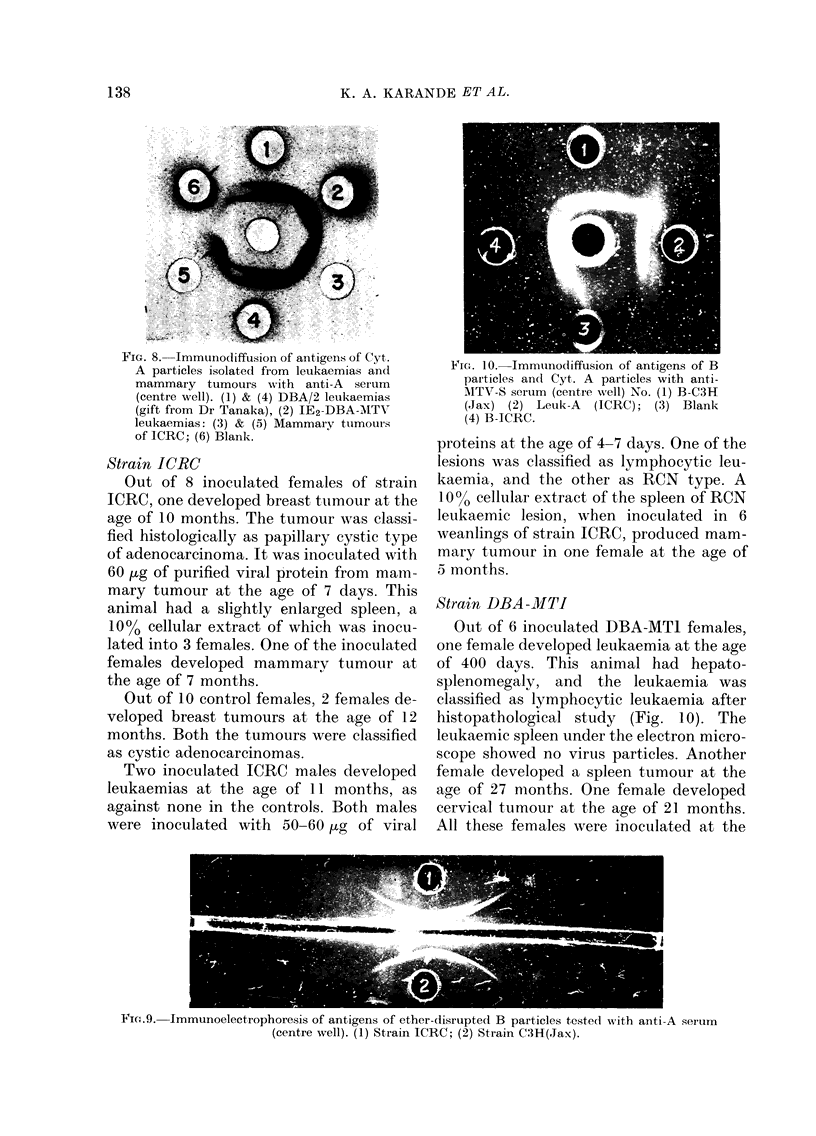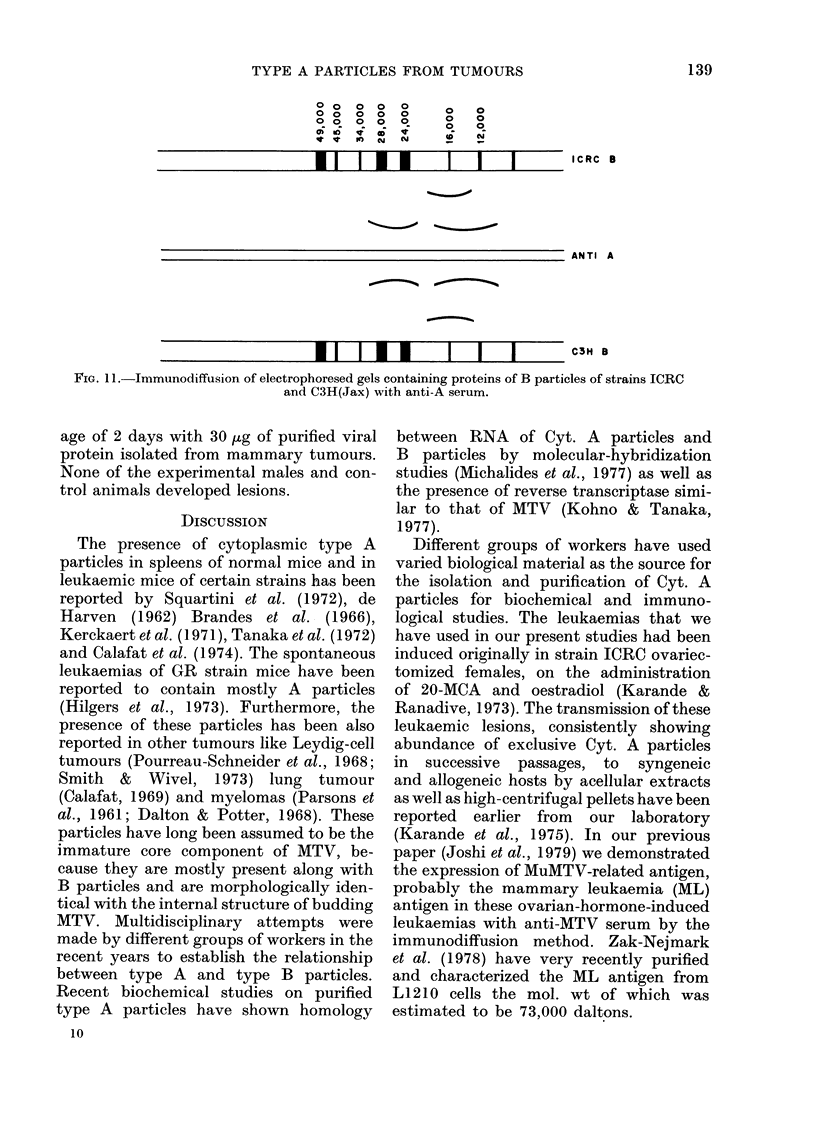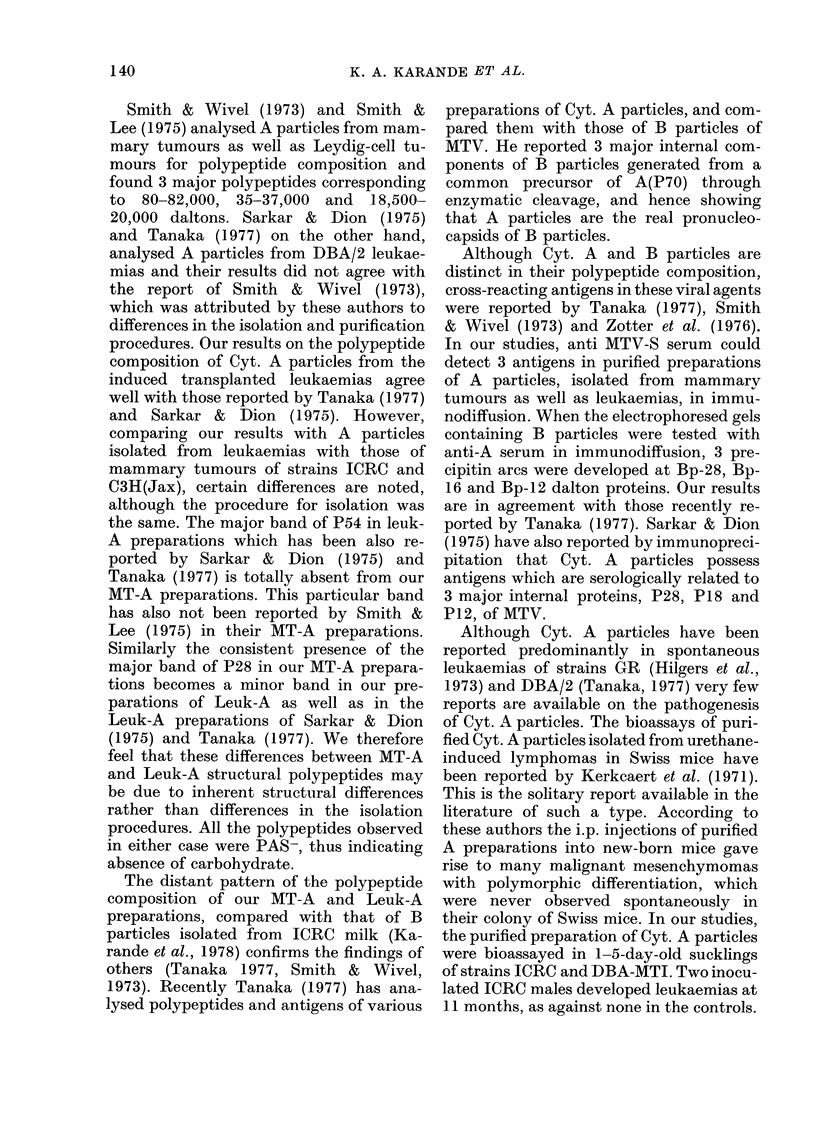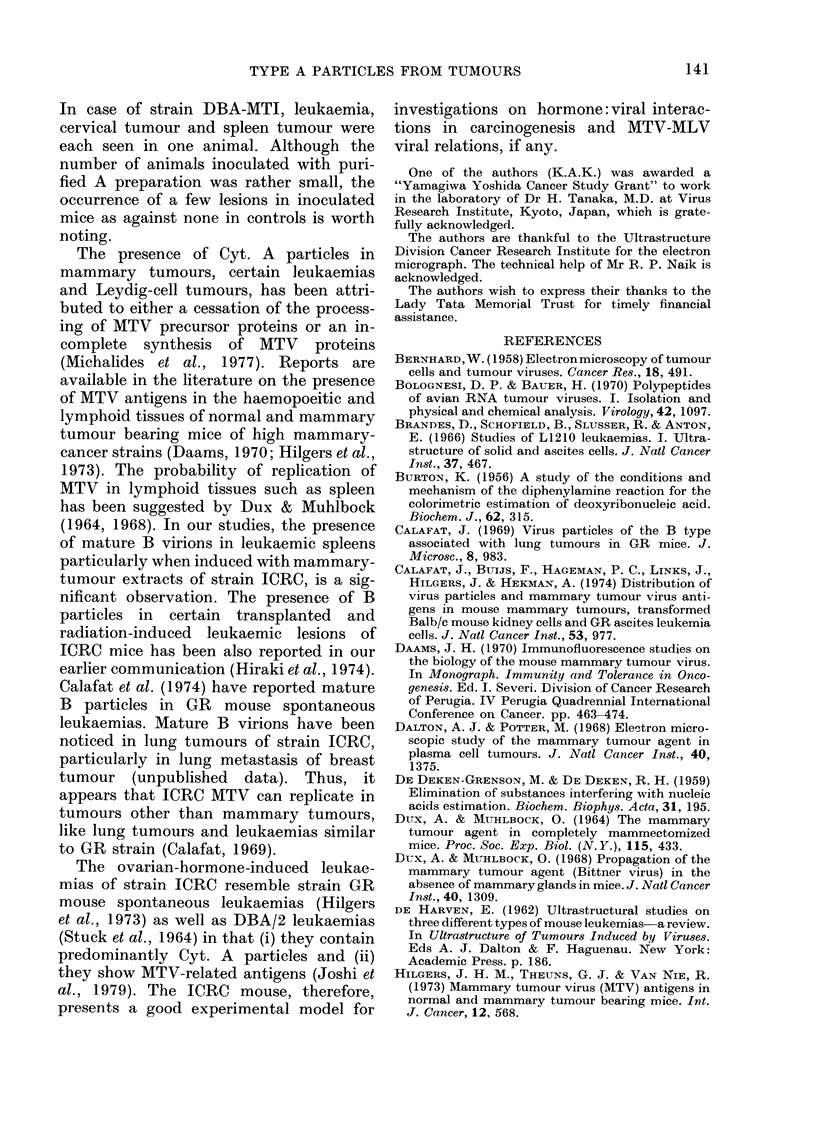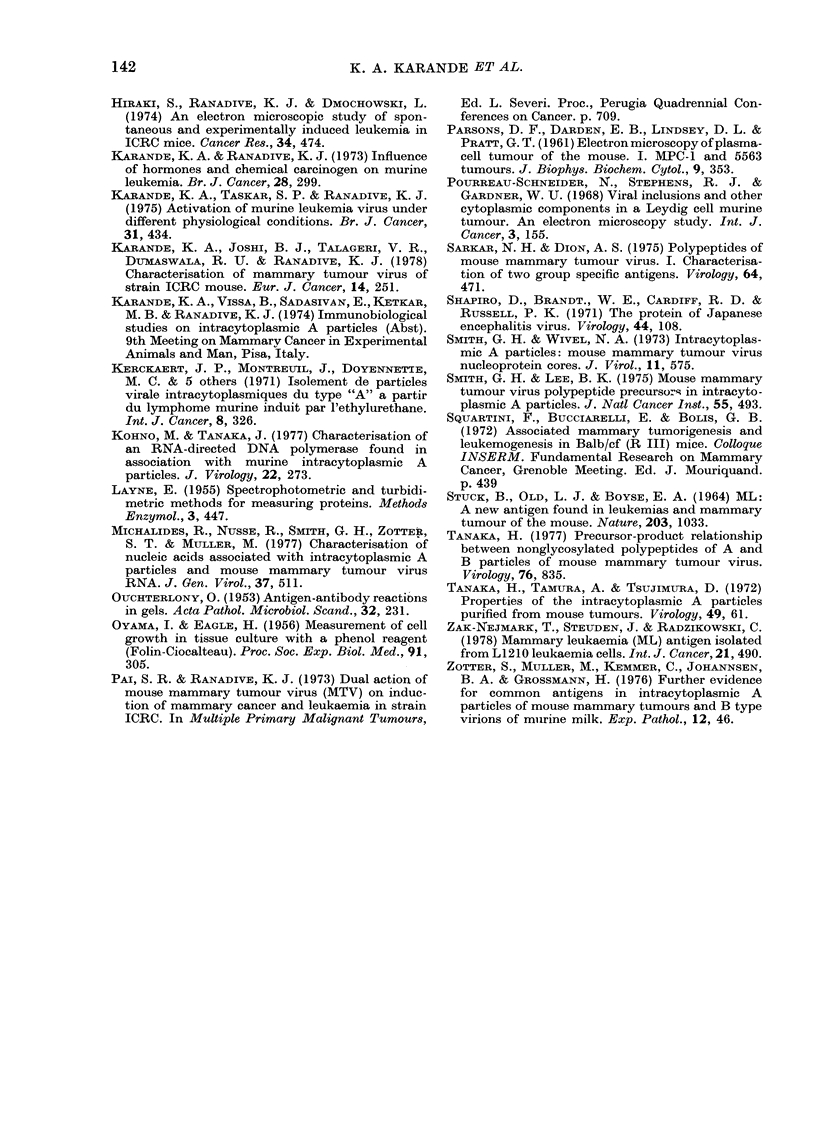# Intracytoplasmic type A particles from mammary tumours and leukaemias of strain ICRC mice.

**DOI:** 10.1038/bjc.1979.23

**Published:** 1979-02

**Authors:** K. A. Karande, B. J. Joshi, V. R. Talageri, R. U. Dumaswala, K. J. Ranadive

## Abstract

**Images:**


*Br. J. Cancer* (1979) **39**, 132

### INTRACYTOPLASMIC TYPE A PARTICLES FROM MAMMARY TUMOURS AND LEUKAEMIAS OF STRAIN ICRC MICE

K. A. KARANDE, B. J. JOSHI, V. R. TALAGERI, R. U. DUMASWALA AND K. J. RANADIVE

*From the Biology Division, Cancer Research Institute, Tata Memorial Centre, Parel, Bombay* 400 012, *India*

Received 11 September 1978 Accepted 2 November 1978

**Summary.—The ovarian-hormone-induced leukaemias of strain ICRC mice, with an abundance of intracytoplasmic type A particles in primary as well as transplanted lesions, were used to study morphological, biophysical, immunological and structural characteristics of type A particles. Mammary tumours of strains ICRC and C3H(Jax) were also used as sources for type A particles. The purified virions banded at the density of 1·20 g/ml in 12–60% linear sucrose-density gradient when subjected to spinning at 113,000 *g* for 4 h. The SDS-polyacrylamide-gel electrophoresis of type A particles from mammary tumours and leukaemias reproducibly resolved at least 8 polypeptides, 2 of these 54,000 and 24,000 dalton proteins, showing variable expression. Type A particles and B particles, despite the fact that each had a distinct polypeptide pattern, showed common antigens with different electrophoretic mobilities. Proteins of 24,000, 18,000 and 12,000 daltons from B particles were found to be antigenically related to those from type A particles. The bioassay studies carried out with purified A particles showed that 2/7 males of strain ICRC and 1/6 females of strain DBA-MTI developed leukaemias, as against none in the controls, when inoculated between the ages of 1–7 days. Spleen tumour and cervical tumour were seen in one female each of strain DBA-MTI.**

STUDIES on the expression and activation of leukaemia virus after the administration of ovarian and pituitary hormones with 20-methylcholanthrene (MCA) in strain ICRC, have been reported from our laboratory (Karande & Ranadive, 1973). The leukaemias induced with 20-MCA plus oestradiol consistently showed abundance of intracytoplasmic type A particles in the leukaemic tissues when studied under the electron microscope (Hiraki *et al.*, 1974; Karande *et al.*, 1975). It was then thought that this was perhaps due to the rapid replication of latent mammary-tumour virus (MTV) in lymphoid cells under suitable conditions. This particular leukaemic line, hereafter referred to as $IE_2$, has been kept in serial transplantation in syngeneic and allogeneic mice through acellular extracts. The presence of predominantly A particles, with rare C particles, persists in these transplanted leukaemic tissues. MTV-related antigens were reported in these leukaemias by immunodiffusion studies (Karande *et al.*, 1974; Joshi *et al.*, submitted) and not C-related antigens. A particles are also seen in large numbers in the mammary tumours of ICRC mice. The development of leukaemias on inoculation of either cellular or acellular extracts of mammary tumours of strain ICRC is noteworthy (Pai & Ranadive, 1973). The cytoplasmic type A (Cyt. A) particle is thought to be the pronucleocapside of the mature B particles of mouse mammary-tumour virus (Tanaka *et al.*, 1972). It was, therefore, thought worthwhile to characterize the Cyt. A particles found in abundance in transplanted leukaemic lesions of $IE_2$ line and mammary tumours of strain ICRC. Since, in recent years, the methods have been standardized for the isolation and purification of Cyt. A particles from tissues (Kerckaert *et al.*, 1971; Tanaka *et al.*, 1972; Smith & Wivel, 1973), it has become possible to carry out different studies on the purified viral material. The present paper describes morphological, immunological and structural characteristics of Cyt. A particles from the induced leukaemic lesions and mammary tumours of strain ICRC.

### MATERIALS AND METHODS

The $IE_2$ leukaemic line has been kept in serial transplantation in strain ICRC and DBA-MTI through cell-free extracts (Karande *et al.*, 1975). Type A particles were isolated and purified from $IE_2$ leukaemic line and mammary tumours of strains ICRC and C3H(Jax) according to the method of Tanaka (1977) which is an improved version of his original method reported in 1972 (Tanaka *et al.*, 1972). B particles were isolated and purified from the milk of ICRC breeders as reported earlier (Karande *et al.*, 1978).

*Antisera.*—Standard anti-A serum (rabbit anti-serum against purified Cyt. A particles from DBA/2 leukaemias) and standard anti-MTV serum (rabbit anti-serum against DBA/2 MTV) were kindly supplied by Dr H. Tanaka (Virus Research Institute, Kyoto, Japan).

The anti-A serum was absorbed with lyophilized tissue powder from strain C57BL mice by incubation at 4°C overnight and then centrifuged at 2,000,000 *g* for 1 h. The anti-B serum was absorbed with 2 volumes of whole milk from C57BL mice to 1 volume of serum by incubation at 37°C for 30 min, storage at 4°C overnight and finally clarified at 200,000 *g* for 30 min. The serum was also absorbed with 50 mg/ml of lyophilized tissue extract from C57BL mice by incubation at 37°C for 1 h and finally clarified at 200,000 *g* for 30 min.

*Immunodiffusion tests and immunoelectrophoresis.*—Double-diffusion tests (Ouchterlony, 1953) using 0·7% agar in normal physiological saline containing 0·001% thiomersal was used. Before testing, purified preparations of Cyt. A particles were treated with 1/10 volume of 1% sodium dodecyl sulphate, whereas the purified B particles were disrupted with ether. Slides were incubated at room temperature in a humidified chamber for 72 h.

Immunoelectrophoresis was performed in 1% Noble agar in barbital buffer (pH 8·6). After the addition of antigens, the electrophoresis was performed at 5 mA/slide for 90 min. After adding the antiserum, slides were kept in a humidified chamber until lines developed.

*Chemical analysis.*—The relative proportion of protein and RNA was ascertained by the measurement of O.D. at 280 and 260 nm (Layne, 1955). The protein content was also measured by the method of Oyama & Eagle (1956), a modification of Lowry's method. The RNA was estimated by the method of De Deken-Grenson & De Deken (1959) whereas DNA was estimated by the method of Burton (1956).

*Polyacrylamide-gel electrophoresis.*—Sodium dodecyl sulphate (SDS) polyacrylamide-gel electrophoresis was performed by method of Shapiro *et al.* (1971). Proteins were precipitated in cold 10% trichloroacetic acid (TCA) for 15 min, and then centrifuged at low speed for 15 min. The pellets were thoroughly drained, washed with 5% TCA and resuspended in 100 $\mu$l of 0·01M sodium phosphate buffer (pH 7·8) containing 1% SDS and 1% $\beta$-mercaptoethanol and incubated at 37°C for 2 h. After complete denaturation of the proteins, the samples were mixed with 100 $\mu$l of 50% sucrose and 5–10 $\mu$l tracking dye (0·25% pyronin) mixed thoroughly and applied to the gels. The gels contained 7·5% acrylamide, 0·25% bis acrylamide, 0·1% SDS and 0·1M sodium phosphate (pH 7·8). Electrophoresis was performed at 3·5 mA/gel for 30 min, followed by 3–3½ h at 7 mA/gel. The gels were stained for 3–4 h in 0·25% Coomassie Brilliant Blue in 40% methanol and 10% glacial acetic acid, and destained by diffusion in 7% acetic acid and 5% methanol. The stained gels were scanned at 580 nm on a Densicord (Photovolt Corporation, N.Y.).

The gels were also stained by periodic-acid-Schiff staining (Bolognesi & Bauer, 1970) for glycoproteins. Molecular weights of the viral polypeptides were estimated from their relative migration in gels compared to standard proteins electrophoresed in parallel gels. The mol. wt standards used were bovine serum albumin (68,000), ovalbumin (43,000), pepsin (35,000), trypsin (23,000), haemoglobin (15,000), lysozyme (14,400) and cytochrome C (11,700).

*Bioassays.*—The pellet of intracytoplasmic type A particles was suspended in PBS, and 0·1 ml of suspension was inoculated into 1–4-days-old suckling mice of strains ICRC and DBA-MTI.

The animals were killed when they were weak and emaciated. The tissues were fixed in 10% formalin and stained with haematoxylin and eosin.

### RESULTS

### *Transplantation of $IE_2$ leukaemias*

The incidence of leukaemia in transplanted animals of strains ICRC and DBA-MTI was high, ranging from 80–90%. The latent period was about one month. These leukaemic lesions showed predominance of cytoplasmic type A particles under the electron microscope (Fig. 1).

Those animals which did not develop leukaemias invariably developed mammary tumours at the age of 12 months. These mammary tumours, whenever inoculated as cell suspensions or as cell-free filtrates in ICRC weanlings, induced leukaemias. Some of these leukaemic lesions exhibited the presence of mature B particles under the electron microscope (Fig. 2).

### *Buoyant density*

The buoyant density of intracytoplasmic type A particles has been reported as 1·26–1·28 g/ml in sucrose-density gradients when they are subjected to spinning for 20 h at 115,000 *g* (Tanaka *et al.*, 1972; Smith & Wivel, 1972). Since the present method is based on rate zonal centrifugation, the position of the virus band in the gradient varies with time. Under the present conditions, the Cyt. A particles banded at 1·16 g/ml when the gradient was subjected to spinning for 1 h at 113,000 *g*. After 4 h spinning, the virus band moved down to 1·20 g/ml (Fig. 3). When the gradient was spun at 172,644 *g* for 5 h, the particles banded at 1·24 g/ml. Cyt. A particles from leukaemic tissues as well as mammary tumours have the same buoyant density in the sucrose gradient.

### *Electron microscopy*

The viral pellets, under the electron microscope, consisted of classical structure of Cyt. A particles as described by Bernhard (1958); 2 concentric ring-like structures with diameter ranging between 500 and 700 Å (Fig. 4).

FIG. 1.—Electron micrograph of spleen of strain ICRC female ($IE_2$ leukaemia), showing patches of cytoplasmic A particles. ×22,520

FIG. 2.—Electron micrograph of mesenteric lymph node of strain ICRC female inoculated with 10% cell suspension of mammary-tumour cells, showing mature B as well as cytoplasmic A particles. ×39,240

FIG. 3.—Density-gradient profile of cytoplasmic A particles from mammary tumours of strain ICRC after 240 min centrifugation at 30,000 rev/min in SW 40 rotor in 12–60% sucrose gradients.

*Chemical composition*

Cyt. A particles isolated both from mammary tumours and leukaemic tissues showed maximum absorption at 260 nm. An average 280/260 ratio was 0·81, which corresponds approximately to 6·5% nucleic acid content. Values obtained by chemical analysis were comparable. DNA was absent from all the preparations of the purified A particles from either source.

*Polypeptide composition* (Refer Table)

Coomassie-blue-stained gels (SDS-PAGE) of A particles from mammary tumours (MT-A) produced 7–8 bands corresponding to 14,000 (P14), 18,000 (P18), 28,000 (P28), 36,000 (P36), 46,000 (P46), 61,500 (P61·5), 70,000 (P70) and 100,000 (P100) daltons (Fig. 5) and for A particles from leukaemic tissues (Leuk-A) 10,000

FIG. 4.—Electron micrograph of the viral band of Fig. 3, showing purified cytoplasmic type A particles.

TABLE.—*Polypeptides of intracytoplasmic type A particles from mammary tumours of strains C3H(Jax) and ICRC and leukaemias of strain ICRC*

| Polypeptide Band No. | Mammary Mol. wt ($\times 10^3$) | Range | % protein | Leukaemias Mol. wt ($\times 10^3$) | Range | % protein |
|---|---|---|---|---|---|---|
| 1 | 14 | 13–14·5 | 2 | 10 | 8–12 | 2·5 |
| 2 | 18 | 17–20 | 12 | 16 MB | 14–18 | 13·0 |
| 3 | 28 MB* | 25–30 | 16 | 28 | 24–30 | 1·0 |
| 4 | 34 MB | 32–36 | 27 | 36 MB | 32–38 | 22·0 |
| 5 | 46 | 43–49 | 8 | 42 | 40–45 | 0·5 |
| 6 | 61·5 | 59–63 | 8 | 54 MB | 50–56 | 20·5 |
| 7 | 70 MB | 68–72 | 17 | 63·5 | 60–65 | 1·5 |
| 8 | 100 | 98–102 | 10 | 75 MB | 72–78 | 38·0 |

* MB = Major bands.

(P10), 16,000 (P16), 28,000 (P28), 36,000 (P36), 42,000 (P42), 54,000 (P54), 63,500 (63·5) and 74,000 (P74) daltons (Fig. 6). All the bands in either case were PAS$^-$. The major bands were found in the region of 27,000–75,000 daltons. However, their relative amounts differed greatly between preparations. Most of the MT-A preparations showed polypeptides P28 and P34 as the major structural proteins, whereas P16, P36 and P54 were the major polypeptides in the case of Leuk-A. Co-electrophoresis of A particles with samples of purified MTV consistently failed to show any similarity in the protein patterns (Fig. 7).

FIG. 5.—SDS-PAGE Electrophoresis pattern of purified Cyt. A particles isolated from mammary tumours of strain ICRC and the densitometric tracing of the gel stained with Coomassie blue.

FIG. 6.—SDS-PAGE electrophoresis pattern of purified Cyt. A particles isolated from leukaemias of strain ICRC and the densitometric tracing of the gel stained with Coomassie blue.

FIG. 7.—SDS-PAGE electrophoresis pattern of purified MuMTV from strain ICRC and the densitometric tracing of the gel stained with Coomassie blue.

*Immunodiffusion and immunoelectrophoresis*

In immunodiffusion, anti-A serum detected 2 precipitin lines with SDS-treated purified preparations of A particles isolated from $IE_2$ leukaemias of strains ICRC and DBA-MTI, as well as from mammary tumours of strain ICRC. The line of identity in Fig. 8 indicates that A particles isolated from leukaemia and mammary tumours have common antigens. The serum, when tested against disrupted B particles isolated from milk of strains ICRC and C3H(Jax), produced one precipitin line with each in immunodiffusion which showed B electrophoretic mobility in immunoelectrophoresis (Fig. 9). However, the serum did not detect any antigens of intact B.

Anti MTV-S serum produced 3 precipitin lines each with disrupted B particles isolated from the milk of strains ICRC and C3H(Jax) and SDS-treated A particles from leukaemias (Fig. 10). The antigens of B particles had $\beta$ electrophoretic characteristics, whereas antigens of A particles showed $\alpha_1$ mobility in immunoelectrophoresis.

B particles isolated from strains ICRC and C3H(Jax) milk were electrophoresed on the polyacrylamide gels. After embedding the gels in agar, immunodiffusion was done using anti-A serum. Three precipitin arcs were formed at the sites of the proteins of the mol. wts of P12, P16 and P28, thus indicating that these proteins of MTV are antigenically similar to those of A particles (Fig. 11).

*Bioassays*

Purified A virus preparations were inoculated into sucklings of strain ICRC (8 females, 7 males) and DBA-MTI (6 females, 5 males) between the ages of 1–7 days. A Groups of 10 females and 8 males of strain ICRC, and 4 females and 4 males of strain DBA-MTI were kept as controls

FIG. 8.—Immunodiffusion of antigens of Cyt. A particles isolated from leukaemias and mammary tumours with anti-A serum (centre well). (1) & (4) DBA/2 leukaemias (gift from Dr Tanaka), (2) $IE_2$-DBA-MTV leukaemias: (3) & (5) Mammary tumours of ICRC; (6) Blank.

FIG. 10.—Immunodiffusion of antigens of B particles and Cyt. A particles with anti-MTV-S serum (centre well) No. (1) B-C3H (Jax) (2) Leuk-A (ICRC); (3) Blank (4) B-ICRC.

*Strain ICRC*

Out of 8 inoculated females of strain ICRC, one developed breast tumour at the age of 10 months. The tumour was classified histologically as papillary cystic type of adenocarcinoma. It was inoculated with 60 $\mu$g of purified viral protein from mammary tumour at the age of 7 days. This animal had a slightly enlarged spleen, a 10% cellular extract of which was inoculated into 3 females. One of the inoculated females developed mammary tumour at the age of 7 months.

Out of 10 control females, 2 females developed breast tumours at the age of 12 months. Both the tumours were classified as cystic adenocarcinomas.

Two inoculated ICRC males developed leukaemias at the age of 11 months, as against none in the controls. Both males were inoculated with 50–60 $\mu$g of viral proteins at the age of 4–7 days. One of the lesions was classified as lymphocytic leukaemia, and the other as RCN type. A 10% cellular extract of the spleen of RCN leukaemic lesion, when inoculated in 6 weanlings of strain ICRC, produced mammary tumour in one female at the age of 5 months.

*Strain DBA-MTI*

Out of 6 inoculated DBA-MT1 females, one female developed leukaemia at the age of 400 days. This animal had hepatosplenomegaly, and the leukaemia was classified as lymphocytic leukaemia after histopathological study (Fig. 10). The leukaemic spleen under the electron microscope showed no virus particles. Another female developed a spleen tumour at the age of 27 months. One female developed cervical tumour at the age of 21 months. All these females were inoculated at the

FIG. 9.—Immunoelectrophoresis of antigens of ether-disrupted B particles tested with anti-A serum (centre well). (1) Strain ICRC; (2) Strain C3H(Jax).

FIG. 11.—Immunodiffusion of electrophoresed gels containing proteins of B particles of strains ICRC and C3H(Jax) with anti-A serum.

age of 2 days with 30 $\mu$g of purified viral protein isolated from mammary tumours. None of the experimental males and control animals developed lesions.

### DISCUSSION

The presence of cytoplasmic type A particles in spleens of normal mice and in leukaemic mice of certain strains has been reported by Squartini *et al.* (1972), de Harven (1962) Brandes *et al.* (1966), Kerckaert *et al.* (1971), Tanaka *et al.* (1972) and Calafat *et al.* (1974). The spontaneous leukaemias of GR strain mice have been reported to contain mostly A particles (Hilgers *et al.*, 1973). Furthermore, the presence of these particles has been also reported in other tumours like Leydig-cell tumours (Pourreau-Schneider *et al.*, 1968; Smith & Wivel, 1973) lung tumour (Calafat, 1969) and myelomas (Parsons *et al.*, 1961; Dalton & Potter, 1968). These particles have long been assumed to be the immature core component of MTV, because they are mostly present along with B particles and are morphologically identical with the internal structure of budding MTV. Multidisciplinary attempts were made by different groups of workers in the recent years to establish the relationship between type A and type B particles. Recent biochemical studies on purified type A particles have shown homology between RNA of Cyt. A particles and B particles by molecular-hybridization studies (Michalides *et al.*, 1977) as well as the presence of reverse transcriptase similar to that of MTV (Kohno & Tanaka, 1977).

Different groups of workers have used varied biological material as the source for the isolation and purification of Cyt. A particles for biochemical and immunological studies. The leukaemias that we have used in our present studies had been induced originally in strain ICRC ovariectomized females, on the administration of 20-MCA and oestradiol (Karande & Ranadive, 1973). The transmission of these leukaemic lesions, consistently showing abundance of exclusive Cyt. A particles in successive passages, to syngeneic and allogeneic hosts by acellular extracts as well as high-centrifugal pellets have been reported earlier from our laboratory (Karande *et al.*, 1975). In our previous paper (Joshi *et al.*, 1979) we demonstrated the expression of MuMTV-related antigen, probably the mammary leukaemia (ML) antigen in these ovarian-hormone-induced leukaemias with anti-MTV serum by the immunodiffusion method. Zak-Nejmark *et al.* (1978) have very recently purified and characterized the ML antigen from L1210 cells the mol. wt of which was estimated to be 73,000 daltons.

Smith & Wivel (1973) and Smith & Lee (1975) analysed A particles from mammary tumours as well as Leydig-cell tumours for polypeptide composition and found 3 major polypeptides corresponding to 80–82,000, 35–37,000 and 18,500–20,000 daltons. Sarkar & Dion (1975) and Tanaka (1977) on the other hand, analysed A particles from DBA/2 leukaemias and their results did not agree with the report of Smith & Wivel (1973), which was attributed by these authors to differences in the isolation and purification procedures. Our results on the polypeptide composition of Cyt. A particles from the induced transplanted leukaemias agree well with those reported by Tanaka (1977) and Sarkar & Dion (1975). However, comparing our results with A particles isolated from leukaemias with those of mammary tumours of strains ICRC and C3H(Jax), certain differences are noted, although the procedure for isolation was the same. The major band of P54 in leuk-A preparations which has been also reported by Sarkar & Dion (1975) and Tanaka (1977) is totally absent from our MT-A preparations. This particular band has also not been reported by Smith & Lee (1975) in their MT-A preparations. Similarly the consistent presence of the major band of P28 in our MT-A preparations becomes a minor band in our preparations of Leuk-A as well as in the Leuk-A preparations of Sarkar & Dion (1975) and Tanaka (1977). We therefore feel that these differences between MT-A and Leuk-A structural polypeptides may be due to inherent structural differences rather than differences in the isolation procedures. All the polypeptides observed in either case were $PAS^-$, thus indicating absence of carbohydrate.

The distant pattern of the polypeptide composition of our MT-A and Leuk-A preparations, compared with that of B particles isolated from ICRC milk (Karande *et al.*, 1978) confirms the findings of others (Tanaka 1977, Smith & Wivel, 1973). Recently Tanaka (1977) has analysed polypeptides and antigens of various preparations of Cyt. A particles, and compared them with those of B particles of MTV. He reported 3 major internal components of B particles generated from a common precursor of A(P70) through enzymatic cleavage, and hence showing that A particles are the real pronucleocapsids of B particles.

Although Cyt. A and B particles are distinct in their polypeptide composition, cross-reacting antigens in these viral agents were reported by Tanaka (1977), Smith & Wivel (1973) and Zotter *et al.* (1976). In our studies, anti MTV-S serum could detect 3 antigens in purified preparations of A particles, isolated from mammary tumours as well as leukaemias, in immunodiffusion. When the electrophoresed gels containing B particles were tested with anti-A serum in immunodiffusion, 3 precipitin arcs were developed at Bp-28, Bp-16 and Bp-12 dalton proteins. Our results are in agreement with those recently reported by Tanaka (1977). Sarkar & Dion (1975) have also reported by immunoprecipitation that Cyt. A particles possess antigens which are serologically related to 3 major internal proteins, P28, P18 and P12, of MTV.

Although Cyt. A particles have been reported predominantly in spontaneous leukaemias of strains GR (Hilgers *et al.*, 1973) and DBA/2 (Tanaka, 1977) very few reports are available on the pathogenesis of Cyt. A particles. The bioassays of purified Cyt. A particles isolated from urethane-induced lymphomas in Swiss mice have been reported by Kerkcaert *et al.* (1971). This is the solitary report available in the literature of such a type. According to these authors the i.p. injections of purified A preparations into new-born mice gave rise to many malignant mesenchymomas with polymorphic differentiation, which were never observed spontaneously in their colony of Swiss mice. In our studies, the purified preparation of Cyt. A particles were bioassayed in 1–5-day-old sucklings of strains ICRC and DBA-MTI. Two inoculated ICRC males developed leukaemias at 11 months, as against none in the controls.

In case of strain DBA-MTI, leukaemia, cervical tumour and spleen tumour were each seen in one animal. Although the number of animals inoculated with purified A preparation was rather small, the occurrence of a few lesions in inoculated mice as against none in controls is worth noting.

The presence of Cyt. A particles in mammary tumours, certain leukaemias and Leydig-cell tumours, has been attributed to either a cessation of the processing of MTV precursor proteins or an incomplete synthesis of MTV proteins (Michalides *et al.*, 1977). Reports are available in the literature on the presence of MTV antigens in the haemopoeitic and lymphoid tissues of normal and mammary tumour bearing mice of high mammary-cancer strains (Daams, 1970; Hilgers *et al.*, 1973). The probability of replication of MTV in lymphoid tissues such as spleen has been suggested by Dux & Muhlbock (1964, 1968). In our studies, the presence of mature B virions in leukaemic spleens particularly when induced with mammary-tumour extracts of strain ICRC, is a significant observation. The presence of B particles in certain transplanted and radiation-induced leukaemic lesions of ICRC mice has been also reported in our earlier communication (Hiraki *et al.*, 1974). Calafat *et al.* (1974) have reported mature B particles in GR mouse spontaneous leukaemias. Mature B virions have been noticed in lung tumours of strain ICRC, particularly in lung metastasis of breast tumour (unpublished data). Thus, it appears that ICRC MTV can replicate in tumours other than mammary tumours, like lung tumours and leukaemias similar to GR strain (Calafat, 1969).

The ovarian-hormone-induced leukaemias of strain ICRC resemble strain GR mouse spontaneous leukaemias (Hilgers *et al.*, 1973) as well as DBA/2 leukaemias (Stuck *et al.*, 1964) in that (i) they contain predominantly Cyt. A particles and (ii) they show MTV-related antigens (Joshi *et al.*, 1979). The ICRC mouse, therefore, presents a good experimental model for investigations on hormone: viral interactions in carcinogenesis and MTV-MLV viral relations, if any.

One of the authors (K.A.K.) was awarded a "Yamagiwa Yoshida Cancer Study Grant" to work in the laboratory of Dr H. Tanaka, M.D. at Virus Research Institute, Kyoto, Japan, which is gratefully acknowledged.

The authors are thankful to the Ultrastructure Division Cancer Research Institute for the electron micrograph. The technical help of Mr R. P. Naik is acknowledged.

The authors wish to express their thanks to the Lady Tata Memorial Trust for timely financial assistance.

### REFERENCES

BERNHARD, W. (1958) Electron microscopy of tumour cells and tumour viruses. *Cancer Res.*, **18**, 491.

BOLOGNESI, D. P. & BAUER, H. (1970) Polypeptides of avian RNA tumour viruses. I. Isolation and physical and chemical analysis. *Virology*, **42**, 1097.

BRANDES, D., SCHOFIELD, B., SLUSSER, R. & ANTON, E. (1966) Studies of L1210 leukaemias. I. Ultrastructure of solid and ascites cells. *J. Natl Cancer Inst.*, **37**, 467.

BURTON, K. (1956) A study of the conditions and mechanism of the diphenylamine reaction for the colorimetric estimation of deoxyribonucleic acid. *Biochem. J.*, **62**, 315.

CALAFAT, J. (1969) Virus particles of the B type associated with lung tumours in GR mice. *J. Microsc.*, **8**, 983.

CALAFAT, J., BUIJS, F., HAGEMAN, P. C., LINKS, J., HILGERS, J. & HEKMAN, A. (1974) Distribution of virus particles and mammary tumour virus antigens in mouse mammary tumours, transformed Balb/c mouse kidney cells and GR ascites leukemia cells. *J. Natl Cancer Inst.*, **53**, 977.

DAAMS, J. H. (1970) Immunofluorescence studies on the biology of the mouse mammary tumour virus. In *Monograph. Immunity and Tolerance in Oncogenesis*. Ed. I. Severi. Division of Cancer Research of Perugia. IV Perugia Quadrennial International Conference on Cancer. pp. 463–474.

DALTON, A. J. & POTTER, M. (1968) Electron microscopic study of the mammary tumour agent in plasma cell tumours. *J. Natl Cancer Inst.*, **40**, 1375.

DE DEKEN-GRENSON, M. & DE DEKEN, R. H. (1959) Elimination of substances interfering with nucleic acids estimation. *Biochem. Biophys. Acta*, **31**, 195.

DUX, A. & MUHLBOCK, O. (1964) The mammary tumour agent in completely mammectomized mice. *Proc. Soc. Exp. Biol. (N.Y.)*, **115**, 433.

DUX, A. & MUHLBOCK, O. (1968) Propagation of the mammary tumour agent (Bittner virus) in the absence of mammary glands in mice. *J. Natl Cancer Inst.*, **40**, 1309.

DE HARVEN, E. (1962) Ultrastructural studies on three different types of mouse leukemias—a review. In *Ultrastructure of Tumours Induced by Viruses*. Eds A. J. Dalton & F. Haguenau. New York: Academic Press. p. 186.

HILGERS, J. H. M., THEUNS, G. J. & VAN NIE, R. (1973) Mammary tumour virus (MTV) antigens in normal and mammary tumour bearing mice. *Int. J. Cancer*, **12**, 568.

HIRAKI, S., RANADIVE, K. J. & DMOCHOWSKI, L. (1974) An electron microscopic study of spontaneous and experimentally induced leukemia in ICRC mice. *Cancer Res.*, **34**, 474.

KARANDE, K. A. & RANADIVE, K. J. (1973) Influence of hormones and chemical carcinogen on murine leukemia. *Br. J. Cancer*, **28**, 299.

KARANDE, K. A., TASKAR, S. P. & RANADIVE, K. J. (1975) Activation of murine leukemia virus under different physiological conditions. *Br. J. Cancer*, **31**, 434.

KARANDE, K. A., JOSHI, B. J., TALAGERI, V. R., DUMASWALA, R. U. & RANADIVE, K. J. (1978) Characterisation of mammary tumour virus of strain ICRC mouse. *Eur. J. Cancer*, **14**, 251.

KARANDE, K. A., VISSA, B., SADASIVAN, E., KETKAR, M. B. & RANADIVE, K. J. (1974) Immunobiological studies on intracytoplasmic A particles (Abst). 9th Meeting on Mammary Cancer in Experimental Animals and Man, Pisa, Italy.

KERCKAERT, J. P., MONTREUIL, J., DOYENNETTE, M. C. & 5 others (1971) Isolement de particles virale intracytoplasmiques du type "A" a partir du lymphome murine induit par l'ethylurethane. *Int. J. Cancer*, **8**, 326.

KOHNO, M. & TANAKA, J. (1977) Characterisation of an RNA-directed DNA polymerase found in association with murine intracytoplasmic A particles. *J. Virology*, **22**, 273.

LAYNE, E. (1955) Spectrophotometric and turbidimetric methods for measuring proteins. *Methods Enzymol.*, **3**, 447.

MICHALIDES, R., NUSSE, R., SMITH, G. H., ZOTTER, S. T. & MULLER, M. (1977) Characterisation of nucleic acids associated with intracytoplasmic A particles and mouse mammary tumour virus RNA. *J. Gen. Virol.*, **37**, 511.

OUCHTERLONY, O. (1953) Antigen-antibody reactions in gels. *Acta Pathol. Microbiol. Scand.*, **32**, 231.

OYAMA, I. & EAGLE, H. (1956) Measurement of cell growth in tissue culture with a phenol reagent (Folin-Ciocalteau). *Proc. Soc. Exp. Biol. Med.*, **91**, 305.

PAI, S. R. & RANADIVE, K. J. (1973) Dual action of mouse mammary tumour virus (MTV) on induction of mammary cancer and leukaemia in strain ICRC. In *Multiple Primary Malignant Tumours*, Ed. L. Severi. Proc., Perugia Quadrennial Conferences on Cancer. p. 709.

PARSONS, D. F., DARDEN, E. B., LINDSEY, D. L. & PRATT, G. T. (1961) Electron microscopy of plasma-cell tumour of the mouse. I. MPC-1 and 5563 tumours. *J. Biophys. Biochem. Cytol.*, **9**, 353.

POURREAU-SCHNEIDER, N., STEPHENS, R. J. & GARDNER, W. U. (1968) Viral inclusions and other cytoplasmic components in a Leydig cell murine tumour. An electron microscopy study. *Int. J. Cancer*, **3**, 155.

SARKAR, N. H. & DION, A. S. (1975) Polypeptides of mouse mammary tumour virus. I. Characterisation of two group specific antigens. *Virology*, **64**, 471.

SHAPIRO, D., BRANDT., W. E., CARDIFF, R. D. & RUSSELL, P. K. (1971) The protein of Japanese encephalitis virus. *Virology*, **44**, 108.

SMITH, G. H. & WIVEL, N. A. (1973) Intracytoplasmic A particles: mouse mammary tumour virus nucleoprotein cores. *J. Virol.*, **11**, 575.

SMITH, G. H. & LEE, B. K. (1975) Mouse mammary tumour virus polypeptide precursors in intracytoplasmic A particles. *J. Natl Cancer Inst.*, **55**, 493.

SQUARTINI, F., BUCCIARELLI, E. & BOLIS, G. B. (1972) Associated mammary tumorigenesis and leukemogenesis in Balb/cf (R III) mice. *Colloque INSERM*. Fundamental Research on Mammary Cancer, Grenoble Meeting. Ed. J. Mouriquand. p. 439

STUCK, B., OLD, L. J. & BOYSE, E. A. (1964) ML: A new antigen found in leukemias and mammary tumour of the mouse. *Nature*, **203**, 1033.

TANAKA, H. (1977) Precursor-product relationship between nonglycosylated polypeptides of A and B particles of mouse mammary tumour virus. *Virology*, **76**, 835.

TANAKA, H., TAMURA, A. & TSUJIMURA, D. (1972) Properties of the intracytoplasmic A particles purified from mouse tumours. *Virology*, **49**, 61.

ZAK-NEJMARK, T., STEUDEN, J. & RADZIKOWSKI, C. (1978) Mammary leukaemia (ML) antigen isolated from L1210 leukaemia cells. *Int. J. Cancer*, **21**, 490.

ZOTTER, S., MULLER, M., KEMMER, C., JOHANNSEN, B. A. & GROSSMANN, H. (1976) Further evidence for common antigens in intracytoplasmic A particles of mouse mammary tumours and B type virions of murine milk. *Exp. Pathol.*, **12**, 46.